# Comprometimento Cardíaco na Síndrome de Sweet: Um Achado Raro numa Doença Rara

**DOI:** 10.36660/abc.20190249

**Published:** 2020-09-11

**Authors:** Luís Graça-Santos, Katarina Kieselova, Fernando Montenegro-Sá, Joana Guardado, João Morais

**Affiliations:** 1 Departamento de Cardiologia Centro Hospitalar de Leiria Leiria Portugal Departamento de Cardiologia, Centro Hospitalar de Leiria, Leiria - Portugal; 2 Departamento de Dermatologia Centro Hospitalar de Leiri Leiria Portugal Departamento de Dermatologia, Centro Hospitalar de Leiria, Leiria - Portugal

**Keywords:** Síndrome de Sweet/fisiopatologia, Eritema Multiforme, Neutrófilos, Miocardite, Corticosteróides/uso terapêutico

## Introdução

A Síndroma de Sweet (SS) é uma dermatose neutrofílica aguda febril caraterizada pela associação de febre, neutrofilia, lesões cutâneas eritematosas moles (pápulas, nódulos, placas), com histologia consistindo predominantemente em neutrófilos maduros localizados na derme superior.^1^ É uma condição rara com distribuição mundial e três formas de apresentação diferentes: idiopática; associada a malignidade e induzida por drogas.^1 - 3^ Manifestações extracutâneas podem ocorrer, mas o comprometimento cardiovascular é extremamente raro.^1 , 2^

## Caso clínico

Homem de 41 anos procurou o serviço de urgência por febre ligeira e lesões cutâneas em agravamento desde há 48 horas. O paciente negou o uso de drogas, alergias conhecidas, histórico pessoal ou familiar de doença relevante, assim como contexto epidemiológico suspeito.

**Figura 1:**
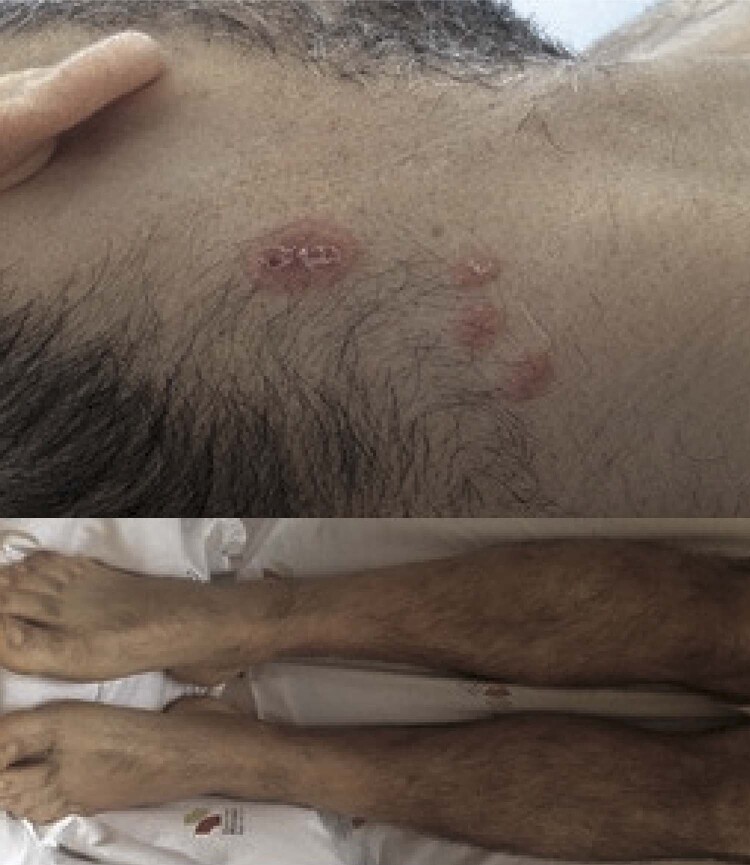
– Lesões cutâneas. Pápulas e placas eritematosas, pseudovesiculadas e dolorosas na nuca (topo); nódulos subcutâneos eritematosos e dolorosos nas pernas (inferior).

O paciente apresentou-se febril (38,3º) e a inspeção revelou pápulas e placas eritematosas, pseudovesiculadas e dolorosas na nuca, pescoço, ombros e braços, assim como nódulos subcutâneos eritematosos e dolorosos (tipo eritema nodoso) nas pernas ( Figura 1 ). Restante exame objetivo sem alterações. O estudo laboratorial mostrou leucocitose ligeira (10800/uL) com 81,4% de neutrófilos, velocidade de sedimentação 89 mm/h (valor normal (N) <10) e proteína-C reactiva (PCR) 128,5 mg/L (N<5,0). Os eletrólitos e os testes de função hepática e renal eram normais.

**Figura 2:**
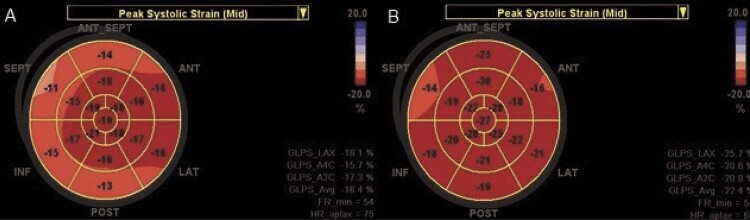
– Strain longitudinal global e segmentar; representação “olho-de-boi” (General Electric®). A) À admissão, o strain global está reduzido (-16,4%); B) Três meses após o tratamento com corticosteroides, o valor normalizou (-22,4%).

Poucas horas após a admissão, o paciente manifestou desconforto torácico ligeiro em repouso. O eletrocardiograma mostrou ritmo sinusal a 58 por minuto com bloqueio atrioventricular do 1º grau e bloqueio incompleto do ramo direito. A troponina I (TnI) foi 1,89ng/mL (N<0,05) e aumentou para 10,82ng/mL após seis horas. O segundo eletrocardiograma mostrou-se sobreponível. O ecocardiograma transtorácico (ETT) foi normal e demonstrou fração de ejeção ventricular esquerda preservada (FEVE; 53% pelo método Simpson biplano) sem alterações da cinética segmentar. Contudo, o *strain* longitudinal global (SLG) mostrou-se reduzido, especialmente à conta dos segmentos médio-basais, com os apicais relativamente poupados ( Figura 2.A ). A angiografia coronariana excluiu doença arterial coronariana (DAC) obstrutiva.

**Figura 3:**
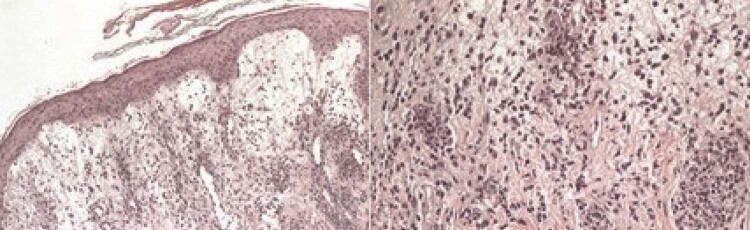
– Histologia da lesão cutânea cervical (coloração hematoxilina-eosina). Edema e infiltrado com predomínio de neutrófilos na derme (esquerda); Ampliação da região da derme mostrando linfócitos, histiócitos e ausência de vasculite (direita).

O paciente foi internado com o diagnóstico presuntivo de dermatose neutrofílica aguda febril. No segundo dia (D2), foi realizada biópsia cutânea e iniciada prednisolona (PDN) oral 1mg/kg/dia tendo em conta a persistência da febre e das lesões cutâneas assim como o aumento da PCR (242 mg/L). Apesar do alívio total da dor torácica, a TnI subiu para 15,01 ng/mL no D2. Após o início de PDN, o paciente permaneceu apirético e os marcadores de inflamação e de necrose miocárdica regrediram. Testes laboratoriais complementares (proteinograma eletroforético, testes de autoimunidade, hormônios tireoidianos, hemoculturas, serologias) revelaram-se normais. A análise histológica da pele mostrou edema subepitelial, infiltrado inflamatório na derme com predomínio polimorfonuclear, e ausência de vasculite ( Figura 3 ). Desse modo, os critérios para o diagnóstico de SS^3^ foram satisfeitos e considerou-se elevada probabilidade de comprometimento cardiovascular na forma de miocardite aguda (MA).^3^ Até D9, o paciente permaneceu apirético, as lesões cutâneas quase cicatrizaram, e os níveis de PCR e TnI diminuíram (9,9 mg/L e 0,32 ng/dL respectivamente). Recebeu alta hospitalar sob PDN em redução progressiva.

**Figura 4:**
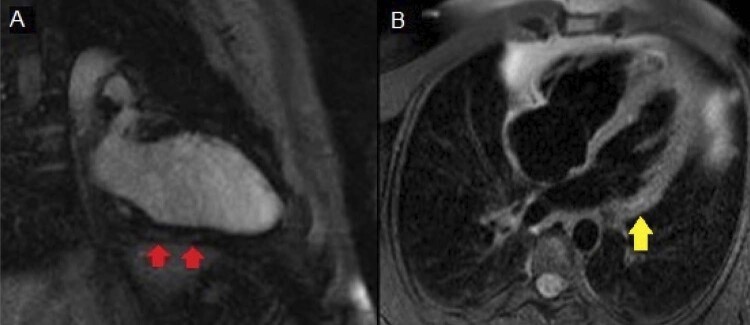
– Ressonância magnética cardíaca. A) Ponderação T1 mostrando ligeiro realce tardio de gadolínio na porção basal da parede inferior (setas vermelhas); B) Ponderação T2 apresentando regiões focais de intensidade de sinal aumentada sugestivas de discreto edema da parede inferolateral (seta amarela).

Após quatro dias, o paciente apresentou-se completamente assintomático, sem lesões cutâneas e ambos os marcadores de inflamação e de necrose miocárdica normalizaram. No sexto dia após a alta, a ressonância magnética cardíaca (RMC) mostrou achados sugestivos de miocardite ( Figura 4 ). A FEVE e SLG melhoraram para 63% e -22,4%, respectivamente, três meses após o tratamento ( Figura 2.B ). O paciente não desejou submeter-se a nova RMC.

Durante dois anos de seguimento, o paciente permaneceu assintomático e sem sinais ou sintomas de doença cardiovascular ou de malignidade.

## Discussão

Os autores apresentam um caso no qual o diagnóstico de SS foi estabelecido pela presença de 2 critérios major e dois minor dos propostos por Driesch.^4^ O tipo idiopático foi assumido devido à ausência de patologia maligna e do uso prévio de quaisquer fármacos. As manifestações extracutâneas podem ocorrer, particularmente em associação com malignidade.^1 , 2^ O comprometimento cardiovascular é extremamente raro e, à luz do nosso conhecimento, apenas dois casos de miocardite foram reportados na variante idiopática.^2 , 5 , 6^ Ambas as manifestações tipicamente respondem aos corticosteroides.^1^

No caso descrito, o desconforto torácico transitório associado à elevação da TnI levantou a suspeita de comprometimento cardiovascular. Quer a MA como o infarto agudo do miocárdio estão descritos como manisfestações cardiovasculares.^2^ A angiografia coronariana, que se mantém como o *gold standard* para o diagnóstico de DAC^7^ ou para sua exclusão na suspeita de MA,^8^ revelou-se normal. Existe alguma evidência de que a ecocardiografia bidimensional *speckle tracking* (2D-EST) pode ajudar a suportar o diagnóstico de MA uma vez que o *strain* longitudinal se correlaciona com a presença de fibrose e edema na RMC e de infiltração linfocitária na biópsia endomiocárdica (BEM).^9 - 12^ No caso relatado, a presença de SLG reduzido sobretudo à custa dos segmentos médio-basais, ao invés dos médio-apicais (padrão típico de DAC significativa),^13^ e a rápida resposta aos corticosteroides reforçaram a probabilidade de MA. Devido à estabilidade clínica e às conhecidas limitações da BEM, foi realizada RMC que sugeriu este diagnóstico de acordo com os critérios Lake-Louise.^8 , 12^ De fato, a RMC emergiu como ferramenta diagnóstica não invasiva e há evidência crescente de que novas técnicas, como o *T1* e *T2 mapping* , podem melhorar a acuidade diagnóstica para MA e também ajudar na monitorização da doença.^8 , 14 , 15^ Adicionalmente, o SLG normalizou três meses após o tratamento, enquanto o paciente permanecia assintomático.

## Mensagens finais

O caso que apresentamos dá ênfase à importância de reconhecer a SS como uma rara mas possível causa de doença cardiovascular, entidade que deve ser precocemente identificada de modo a iniciar tratamento adequado.

No caso presente, o diagnóstico de MA foi altamente sugerido por modalidades imagiológicas não invasivas após a exclusão de DAC obstrutiva. À luz do nosso conhecimento, essa foi a primeira vez que a RMC foi usada para avaliar comprometimento miocárdico num doente com SS e também a primeira em que a análise por 2D-EST foi usada na monitorização evolutiva. Ambas as manifestações, cutâneas e cardiovasculares, regrediram completamente após tratamento com corticosteroides.
