# Supplementary material for: Comprometimento Cardíaco na Síndrome de Sweet: Um Achado Raro numa Doença Rara
Source: Arq Bras Cardiol. 2020 Sep 11;115(1 Suppl 1):6–9. [Article in Portuguese] doi: 10.36660/abc.20190249 (PMC8386950; doi:10.36660/abc.20190249)
Supplement: Translation

# Translation: Myocardial Involvement in Sweet Syndrome: A Rare Finding in a Rare Condition

**Authors:** Luís Graça-Santos, Katarina Kieselova, Fernando Montenegro-Sá, Joana Guardado, João Morais

**Affiliations:** 1 Portugal Department of Cardiology, Leiria Hospital Centre, Leiria - Portugal; 2 Portugal Department of Dermatology, Leiria Hospital Centre, Leiria - Portugal

**Keywords:** Sweet Syndrome/physiopathology, Erythema Multiforme, Neutrophils, Myocarditis, Adrenal Cortex Hormones/therapeutic use

## Introduction

Sweet Syndrome is an acute febrile neutrophilic dermatosis characterized by an association of fever, neutrophilia, tender erythematous skin lesions (papules, nodules, and plaques), as well as pathologic findings consisting predominantly of mature neutrophils typically located in the upper dermis.^1^ It is a rare condition with a worldwide distribution which can present as one of three main clinical types: idiopathic, malignancy-associated, or drug-induced.^1 - 3^ Extracutaneous manifestations may occur but cardiovascular involvement is extremely rare.^1 , 2^

## Case presentation

A previously healthy 41-year-old male presents to the emergency department with a 48-hour history of mild fever and worsening widespread skin lesions. He denied recent drug intake, known allergies, relevant personal or familial diseases, as well as suspicious epidemiological context.

The patient was febrile (38.3ºC) and heart rate, blood pressure, and oxygen saturation were all normal. The chest and abdominal examination were both unremarkable. Skin examination revealed painful pseudovesiculate, erythematous papules, and plaques on the nape, neck, shoulders, and arms, as well as painful hyperpigmented subcutaneous nodules ( *erythema nodosum-like* ) on the legs ( Figure 1 ). Blood tests showed slight leucocytosis (10800/uL) with 81.4% of neutrophils, erythrocyte sedimentation rate was 89mm/h (normal value (NV) <10) and C-reactive protein (CRP) 128.5mg/L (NV<5.0). Electrolytes, renal and hepatic profiles were normal.

**Figure 1:** – Skin examination. Pseudovesiculate, erythematous papules and plaques on the nape (top); hyperpigmented subcutaneous nodules on the legs (bottom).

A few hours later, the patient complained of transient chest discomfort at rest. The electrocardiogram showed sinus rhythm at 58 per minute with first-degree atrioventricular block plus incomplete right bundle branch block. Troponin I (TnI) was 1.89ng/mL (NV<0.05) and raised up to 10.82ng/mL six hours later. The repeated electrocardiogram was identical to the previous one. Transthoracic echocardiogram (TTE) was normal, demonstrating preserved left ventricular ejection fraction (LVEF; 53% Simpson’s biplane method) with no major wall motion abnormalities. However, global longitudinal peak systolic strain (GLPSS) was reduced, especially at the expense of the mid-basal segments being the apex relatively spared ( Figure 2.A ). Coronary angiogram excluded obstructive coronary artery disease (CAD).

**Figure 2:** – Global and segmental longitudinal strain analysis; “bull-eye” plot (General Electric®). (A) Global strain is reduced (-16.4%) at admission; (B) and normalized (-22.4%) three months after corticosteroid treatment.

The patient was admitted to the internal medicine ward with the presumptive diagnosis of acute febrile neutrophilic dermatosis. On the second day (D2), skin biopsy was performed and oral prednisolone (PDN) 1mg/Kg/day was initiated, taking into consideration the persistence of both fever and skin lesions as well as the increase of the CRP value (242mg/L). Despite no chest discomfort relapse, TnI reached a peak level of 15.01ng/mL on D2. After initiating PDN, the patient remained afebrile, and both systemic inflammatory and myocardial injury biomarkers started to decrease. Complementary laboratorial tests (such as electrophoretic proteinogram, autoimmunity screening, thyroid hormones, blood cultures and serology tests) were normal. The histological skin analysis revealed subepithelial oedema, dermal inflammatory infiltrate with polymorphonuclear predominance and absence of vasculitis ( Figure 3 ). Based on this information, diagnostic criteria were fulfilled for SS^3^ and a high likelihood for cardiovascular involvement presenting as acute myocarditis (AM)^3^ was considered. On D9, there had been no fever relapse, skin lesions were mostly healed, and CRP and TnI levels almost normalized (9.9mg/L and 0.32ng/dL respectively). The patient was discharged on a tapering corticosteroid regimen.

**Figure 3:** – Histology of the cervical skin lesion (haematoxylin-eosin stain). Predominant neutrophilic dermal infiltrate and oedema (left). Zoom over the dermal area showing some lymphocytes, histiocytes, and absence of vasculitis (right).

Four days later, the patient presented completely asymptomatic with no skin lesions and both systemic inflammatory and myocardial injury biomarkers have normalized. A cardiovascular magnetic resonance (CMR) was scheduled six days after discharge and showed findings suggestive of myocarditis ( Figure 4 ). Both LVEF and GLPSS improved up to 63% and -22.4%, respectively, three months after the initial assessment ( Figure 2.B ). The patient did not wish to undergo a second CMR study.

**Figure 4:** – Cardiovascular magnetic resonance imaging. (A) T1-weighted image demonstrating patchy subepicardial late gadolinium enhancement (red arrows) in the basal portion of the inferior wall; (B) T2-weighted image presenting focal regions of increased signal intensity (yellow arrow) suggestive of slight oedema in the inferolateral wall.

During a two-year follow-up, the patient remained completely asymptomatic with no signs or symptoms of cardiovascular or malignant disease.

## Discussion

We present a case where the diagnosis of SS was established as two major and three minor Driesch criteria were identified.^4^ The idiopathic type was assumed since no recent drug intake was reported and no signs of malignant disease were present. Extracutaneous manifestations may occur, particularly in association with malignancy.^1 , 2^ Cardiovascular involvement is extremely rare and up to this date, only two cases of myocarditis have been reported in the idiopathic type, to our best knowledge.^2 , 5 , 6^ Both manifestations typically respond well to corticosteroids.^1^

In this patient, the presence of transient chest discomfort associated with TnI elevation raised the suspicion of cardiovascular involvement. Both AM and acute myocardial infarction have been previously described as cardiovascular manifestations.^2^ Coronary angiogram remains the gold standard for the diagnosis of CAD^7^ or for its exclusion in case of suspected AM^8^ and was normal in this case. There is some evidence that two-dimensional speckle tracking echocardiography (2D-STE) may help support the diagnosis of AM since GLPSS correlates with the presence of fibrosis and oedema on CMR and with lymphocytic infiltrates on endomyocardial biopsy (EMB).^9 - 12^ In our case, the presence of GLPSS reduction mainly at the expense of the mid-basal segments, instead of the mid-apical segments (typical pattern of significant CAD),^13^ and the prompt response to corticosteroids raised the likelihood of AM. Due to clinical stability and the well-known limitations of EMB, a CMR was later performed and suggested this diagnosis according to the Lake-Louise criteria.^8 , 12^ In fact, CMR has emerged as a useful non-invasive diagnostic tool and there is growing evidence that novel techniques, such as T1 and T2 mapping, may improve its diagnostic accuracy for myocarditis and help monitor disease evolution.^8 , 14 , 15^ Additionally, GLPSS normalized three months after treatment, while the patient remained asymptomatic.

## Final messages

The case we present emphasizes the importance of acknowledging SS as a rare yet plausible cause of cardiovascular disease, and one that should be early recognized in order to start adequate treatment.

In this case, the diagnosis of AM was highly suggested by the combination of non-invasive imaging modalities after CAD exclusion. To our best knowledge, this was the first time CMR was used to assess myocardial involvement in a patient with SS and also the first to report the use of 2D-STE for evolution monitoring. Both cutaneous and cardiovascular manifestations completely regressed after corticosteroid treatment.
